# Immuno-Modulatory and Anti-Inflammatory Effects of Dihydrogracilin A, a Terpene Derived from the Marine Sponge *Dendrilla membranosa*

**DOI:** 10.3390/ijms18081643

**Published:** 2017-07-28

**Authors:** Elena Ciaglia, Anna Maria Malfitano, Chiara Laezza, Angelo Fontana, Genoveffa Nuzzo, Adele Cutignano, Mario Abate, Marco Pelin, Silvio Sosa, Maurizio Bifulco, Patrizia Gazzerro

**Affiliations:** 1 Department of Medicine, Surgery and Dentistry “Scuola Medica Salernitana”, University of Salerno, Via Salvatore Allende, 84081 Baronissi Salerno, Italy; eciaglia@unisa.it (E.C.); m.abate23@studenti.unisa.it (M.A.); 2 Department of Pharmacy, University of Salerno, Via Giovanni Paolo II, 84084 Fisciano, Salerno, Italy; amalfitano@unisa.it; 3 Department of Biology and Cellular and Molecular Pathology, University of Naples Federico II, Via Pansini, 80131 Naples, Italy; chiara.laezza@cnr.it; 4 Institute of Endocrinology and Experimental Oncology, IEOS CNR, Via Pansini 5, 80131 Naples, Italy; 5 Bio-Organic Chemistry Unit, Institute of Biomolecular Chemistry-CNR, Via Campi Flegrei 34, Pozzuoli, 80131 Naples; Italy, afontana@icb.cnr.it (A.F.); nuzzo.genoveffa@icb.cnr.it (G.N.); acutignano@icb.cnr.it (A.C.); 6 Department of Life Sciences, University of Trieste, 34127 Trieste, Italy; mpelin@units.it (M.P.), ssosa@units.it (S.S.); 7 CORPOREA-Fondazione Idis-Città della Scienza, via Coroglio 104 e 57, 80124 Naples, Italy

**Keywords:** marine sponge, natural compound, inflammation, lymphocytes

## Abstract

We assessed the immunomodulatory and anti-inflammatory effects of 9,11-dihydrogracilin A (DHG), a molecule derived from the Antarctic marine sponge *Dendrilla membranosa*. We used in vitro and in vivo approaches to establish DHG properties. Human peripheral blood mononuclear cells (PBMC) and human keratinocytes cell line (HaCaT cells) were used as in vitro system, whereas a model of murine cutaneous irritation was adopted for in vivo studies. We observed that DHG reduces dose dependently the proliferative response and viability of mitogen stimulated PBMC. In addition, DHG induces apoptosis as revealed by AnnexinV staining and downregulates the phosphorylation of nuclear factor kappa-light-chain-enhancer of activated B cells (NF-κB), signal transducer and activator of transcription (STAT) and extracellular signal–regulated kinase (ERK) at late time points. These effects were accompanied by down-regulation of interleukin 6 (IL-6) production, slight decrease of IL-10 and no inhibition of tumor necrosis factor-alpha (TNF-α) secretion. To assess potential properties of DHG in epidermal inflammation we used HaCaT cells; this compound reduces cell growth, viability and migration. Finally, we adopted for the in vivo study the croton oil-induced ear dermatitis murine model of inflammation. Of note, topical use of DHG significantly decreased mouse ear edema. These results suggest that DHG exerts anti-inflammatory effects and its anti-edema activity in vivo strongly supports its potential therapeutic application in inflammatory cutaneous diseases.

## 1. Introduction

Sponges, seaweeds, snails and soft corals are marine organisms representing an unexploited source of novel compounds with promising application to human wellbeing [1,2]. These metabolites exhibit various bioactivities and potential pharmacological properties, and many of them are currently on the market or clinical trials as anti-cancer, analgesic, immunomodulatory or anti-inflammatory agents [3,4,5,6,7,8,9,10,11]. Among the natural products, one of the most studied groups of molecules is represented by terpenes [12], secondary metabolites formed by repetitions of C5 isoprene units that derive from two distinct biochemical routes named mevalonate or non-mevalonate pathways [13,14,15,16]. Terpenes are generally classified into hemi, mono, sesqui, di, sester, or tri based according to the number of the isoprene units. These compounds have been found largely in higher plants [17,18] and in lower invertebrates including marine organisms [19,20,21,22,23,24]. Marine diterpenoids embrace a diverse and promising class of molecules exhibiting a range of effects including antiviral, antibacterial, antiparasite, anticancer, and anti-inflammatory activity [19,25,26,27,28,29]. Studies suggested that briarane-or cembrane-type diterpenes exert anti-inflammatory activity as they inhibit pro-inflammatory enzymes, such as cyclooxygenase (COX-2) and inducible nitric oxide synthase (iNOS) in murine macrophages activated with lipopolysaccharide (LPS) [30,31,32,33]. Cembranoids such as gibberosenes, grandilobatin, sarcocrassocolides, querciformolides, crassarines, crassumolides, sinularolides, durumolides and columnariols have shown capacity to block the expression of iNOS and/or COX-2 by LPS-activated RAW264.7 cells [34]. Some cembranoids have been identified as modulators of nuclear factor kappa-light-chain-enhancer of activated B cells (NF-κB) signaling pathway [35,36,37]. Indeed, in recent years, anti-inflammatory activity for eunicellin-based diterpenoids isolated from soft corals, has been described [38,39]. Marine diterpene glycosides are characterized by a diterpene aglycone core and a carbohydrate moiety. Among these compounds, glycosides, eleutherobin, fuscosides, and pseudopterosins are the most studied [40]. Eleutherobin is a microtubule-targeted agent currently used in preclinical studies [41,42]. The pseudopterosins have been described as molecules with important anti-inflammatory and analgesic properties [43,44]. Fuscosides A and B when topically applied, decrease phorbol myristate acetate (PMA)-induced edema in mouse ears by blocking neutrophil infiltration. Fuscoside B blocks the synthesis of leukotriene C4 in calcium ionophore-activated murine macrophages [45,46]. Another family of diterpenoids, verticillane-based diterpenoids, have recently demonstrated anti-inflammatory properties, members of this family inhibit iNOS in LPS-stimulated RAW264.7 cells [47]. Furthermore, a tricyclic brominated diterpenoid, the neorogioltriol isolated from algae, inhibited the activation of NF-κB and the secretion of tumor necrosis factor-alpha (TNF-α), nitric oxide (NO), and COX-2 in LPS stimulated macrophages. In an animal model of carrageenan-induced local inflammation, neorogioltriol decreased edema formation [48]. Mollusc derived dolabellane diterpenoids, also isolated from plants, exert antiprotozoa [49], antiviral [50], and antibacterial [51] activities [52]. Dolabelladienetriol has been suggested to have anti-inflammatory properties as downregulates the secretion of TNF-α and NO through the inhibition of NF-κB activation in *Leishmania amazonensis* infected and uninfected macrophages [53].

In a recent work about a novel platform of drug discovery based on an automatic fractionation of marine samples by a simple and versatile protocol of solid-phase extraction [54], we reported that the diterpenoid 9,11-dihydrogracilin (DHG) [55] from the Antarctic sponge *Dendrilla membranosa* stimulated the response of human peripheral blood mononuclear cells (PBMCs). DHG belongs to the spongiane family, a very large class of sponge-derived natural products showing several promising activities [56]. Here, we investigate the immunomodulatory and anti-inflammatory properties of DHG by in vitro and in vivo models. The effects exhibited by this compound support its potential as novel anti-inflammatory drug.

## 2. Results

### 2.1. 9,11-Dihydrogracilin A (DHG)-Mediated Inhibition of Human Peripheral Blood Mononuclear Cells (PBMC) Proliferation and Viability

**Figure 1:**
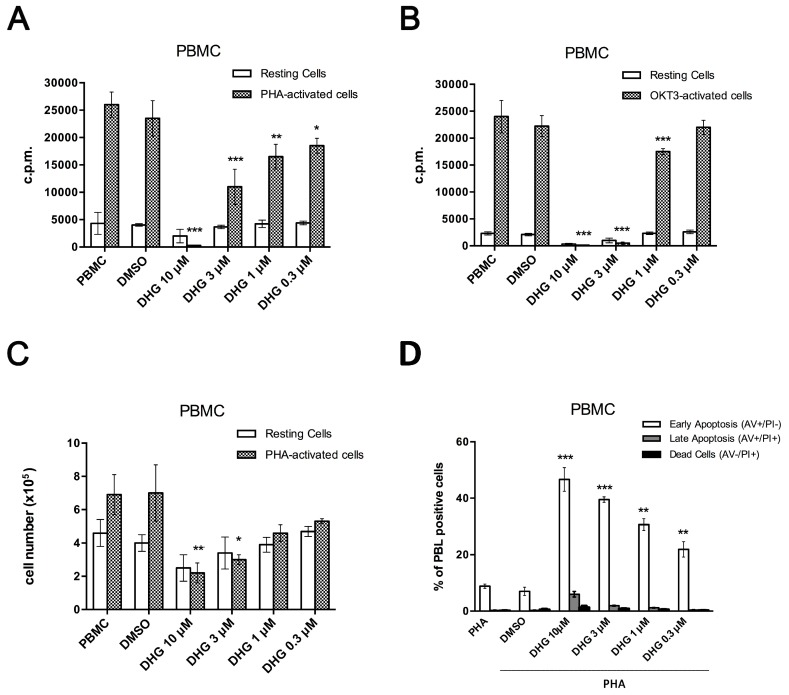
9,11-Dihydrogracilin A (DHG) inhibits Peripheral Blood Mononuclear Cells (PBMC) proliferation and viability and induces apoptosis. (**A**) Unstimulated PBMC and phytohemagglutinin (PHA)-activated PBMC from healthy donors were treated with DHG at the indicated concentrations. Proliferation was measured after 18h of ^3^H-thymidine incorporation (1 µCi). The counts per minutes (c.p.m.) ± the SD of the triplicates of five independent experiments are shown. (ANOVA * *p* < 0.05, *** *p* < 0.001, ** *p* < 0.01 versus PHA-treated PBMC); (**B**) Unstimulated PBMC and CD3 monoclonal antibody (OKT3)-activated PBMC of healthy donors were treated with DHG at the indicated concentrations. Proliferation was measured after 18h of ^3^H-thymidine incorporation (1 µCi). The c.p.m. ± the SD of the triplicates of five independent experiments are shown. (ANOVA * *p* < 0.05, *** *p* < 0.001, ** *p* < 0.01 versus OKT3-treated PBMC); (**C**) Unstimulated PBMC and PHA-activated PBMC from healthy donors were treated with DHG, cultured for 6 days and stained with trypan blue. Cell viability was compared to that observed in PHA-activated PBMC (ANOVA * *p* < 0.05, ** *p* < 0.01). The histogram reported show the percent of live PBMC; (**D**) Induction of apoptosis was measured by annexin V and propidium iodide (PI) double staining through fluorescence-activated cell sorting (FACS) analysis in DHG-treated healthy donor PBMC, after 48 h. The panel reporting representative dot plots of 4 different experiments performed with similar results is included in the supplementary section (Supplementary Figure S1). Histograms in **D** indicate total percentage of early (Annexin V-positive cells/PI-negative cells) and late apoptotic events (Annexin V/PI-double positive cells) as well as necrotic cells (Annexin V-negative cells/PI-positive cells). Results are representative of 4 independent experiments and expressed as mean ± SD (ANOVA, *** *p* < 0.001, ** *p* < 0.01). DMSO, dimethyl sulfoxide.

First we asked if the immuno-modulatory potential of DHG could affect mitosis of CD3 monoclonal antibody (OKT3)- and Phytohemagglutinin (PHA)-activated healthy-PBMC. Proliferation of PBMC was determined after 4 or 6 days of stimulation with OKT3 (1 μg/mL) and PHA (1.5%) respectively, by measuring [^3^H]-thymidine incorporation. As shown in Figure 1A,B, all mitogenic stimuli induced a significant proliferation of PBMC. The co-treatment with DHG at selected concentrations, ranging from 0.3 to 10 µM, resulted in a dose-response inhibition of mitosis of PHA and to a more extent of OKT3-stimulated PBMC. A better dose–response profile was observed using PHA as stimulus, thus for further experiments we used only PHA.

In order to assess whether besides inhibition of DNA synthesis, DHG could affect cell viability of PBMC, we counted the cells after the staining with trypan blue. DHG decreased the number of viable cells in a concentration-dependent manner (Figure 1C), specifically, at 10 µM, it significantly reduced viable cell number of 73 ± 2.4%. Of note the viability of DHG-treated resting cells was not significantly affected, thus excluding its possible toxic effect. Then, to better characterize the nature of cytotoxic effects mediated by DHG in activated PBMC, we next performed cell death assays by Annexin-V and propidium iodide double staining (Supplementary Figure S1). Here, we registered a dose-dependent induction of apoptosis, resulting in the death of 43.1 ± 2.4% of cells already after 48h exposure at the highest dose of 10 µM DHG (Figure 1D).

### 2.2. DHG Effects on Signaling Pathways

**Figure 2:**
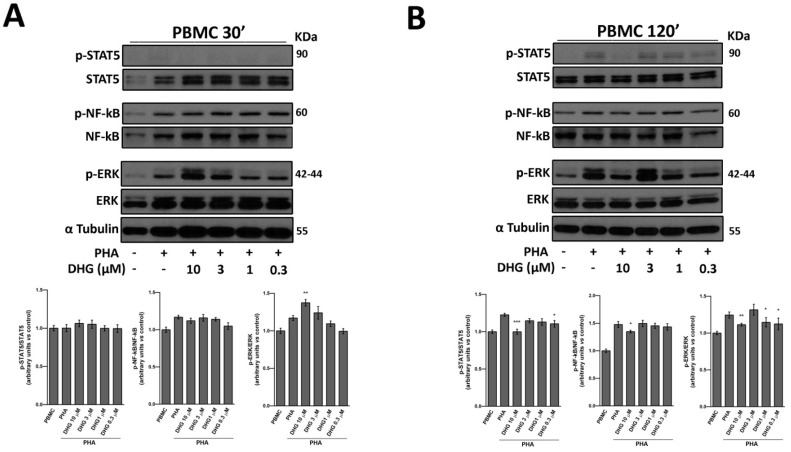
DHG effects on NF-κB, Signal Transducer and Activator of Transcription 5 (STAT5) and Extracellular Signal–regulated Kinase (ERK) phosphorylation. Western blot analysis performed on whole cell extracts from 30 min (**A**) and 120 min (**B**) of culture in the presence and in the absence of DHG at the indicated concentrations. α-tubulin was used as control of protein loading. Panels show representative results from 3 different experiments performed independently. Histograms below represent mean ± SD in densitometry units of scanned immunoblots from the 3 different experiments (ANOVA, *** *p* < 0.001, ** *p* < 0.01, * *p* < 0.05).

Since signal transducer and activator of transcription 5 (STAT5), extracellular signal–regulated kinase (ERK), and NF-κB signaling pathways are critical for PBMC activation following stimulation with PHA, we moved to investigate whether and in which way these signaling events were affected by increasing doses of DHG at early time points. As reported in Figure 2A, ERK was phosphorylated in response to 30 min-PHA stimulation. However, DHG 10 µM led to significantly greater levels of phospho-extracellular signal–regulated kinase (p-ERK) compared with the effect observed in response to the mitogen alone. On the other hand, phospho-nuclear factor kappa-light-chain-enhancer of activated B cells (p-NF-κB) was not affected by DHG treatment. Moreover, no signals were observed in the activation of STAT5 pathway at this early time point. On the contrary, as expected, after in vitro stimulation for 120 min, PHA enhanced tyrosine phosphorylation of STAT5, which is instead significantly inhibited by DHG co-treatment. Similarly, DHG at the highest dose decreased ERK and NF-κB activity, compared to control PHA-activated cells (Figure 2B). Kinetic studies (Supplementary Figure S2) revealed that inhibition of NF-κB phosphorylation by DHG co-treatment at all the concentrations used was still observed after 5 days of stimulation, suggesting a long lasting effect of the compound.

### 2.3. Cytokine Production by DHG-Treated PBMC upon Phytohemagglutinin (PHA) Stimulation

**Figure 3:**
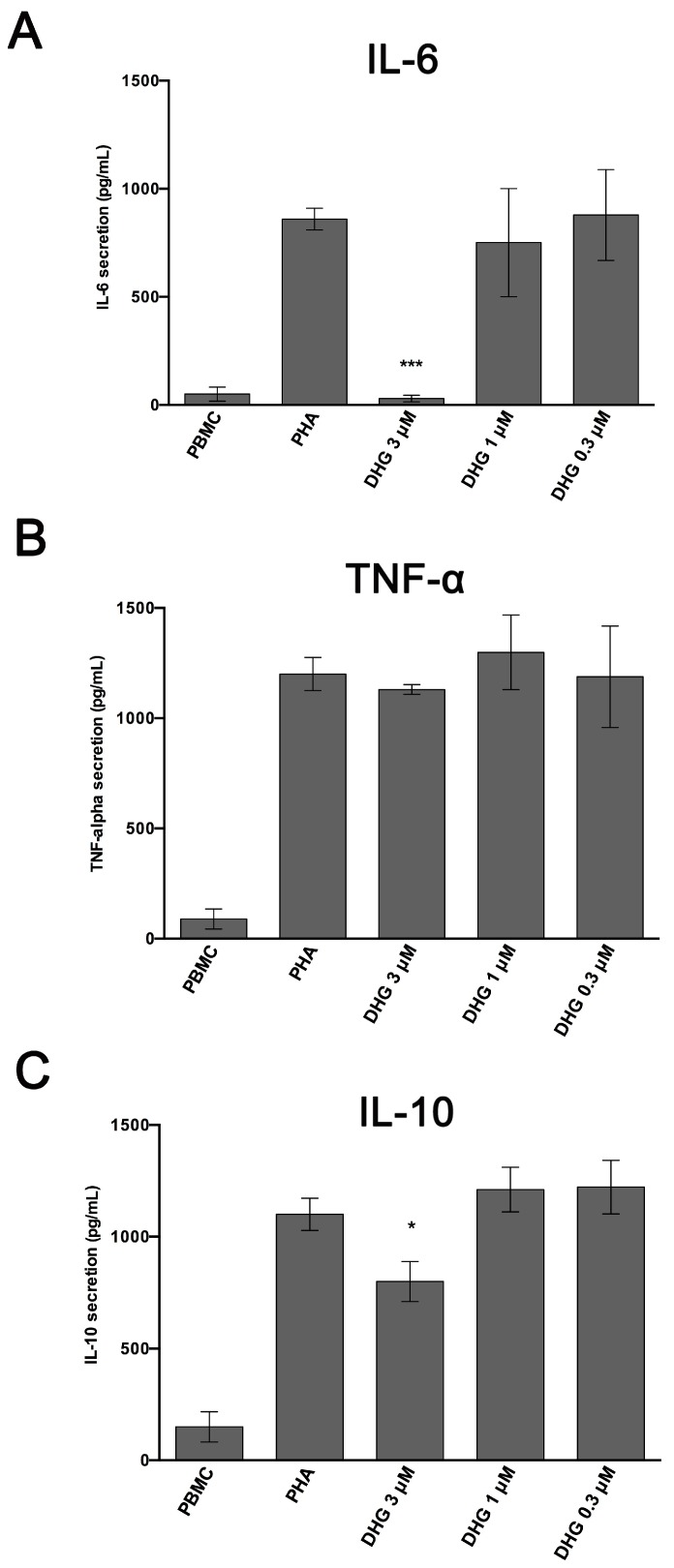
Cytokine secretion profile of DHG-treated PBMC. PBMC of healthy donors (*n* = 4) were activated with PHA (1.5%) for 24 h in the presence and in the absence of DHG at the indicated concentrations. Unstimulated cells are included as control (PBMC) in the figure. Supernatants were harvested and the concentrations of interleukin 6 (IL-6) (**A**), tumor necrosis factor-alpha (TNF-α) (**B**) and IL-10 (**C**) determined by ELISA immunoassay. Values reported refer to mean ± SD of four different donors. Statistical analyses are reported (ANOVA; * *p* < 0.05; *** *p* < 0.001).

After proper activation, PBMC can secrete numerous cytokines, through which they coordinate immune response, such as interleukin 6 (IL-6), TNF-α and IL-10 [57,58]. After 24h of incubation, we then assessed the capacity of DHG-treated PBMC to produce these soluble factors. As expected, stimulation of PBMC by PHA induced secretion of all soluble factors tested. Notably, the treatment with DHG, at the concentration of 3 µM significantly inhibited the production of IL-6 (Figure 3A), without interfering with the levels of the other pro-inflammatory factor TNF-α (Figure 3B). Finally, a slight decrease in the level of the contro-regulatory action of IL-10 was also documented (Figure 3C).

### 2.4. DHG Effects on Activation Marker Surface Expression in T and Natural Killer (NK) Cell Compartments

**Figure 4:**
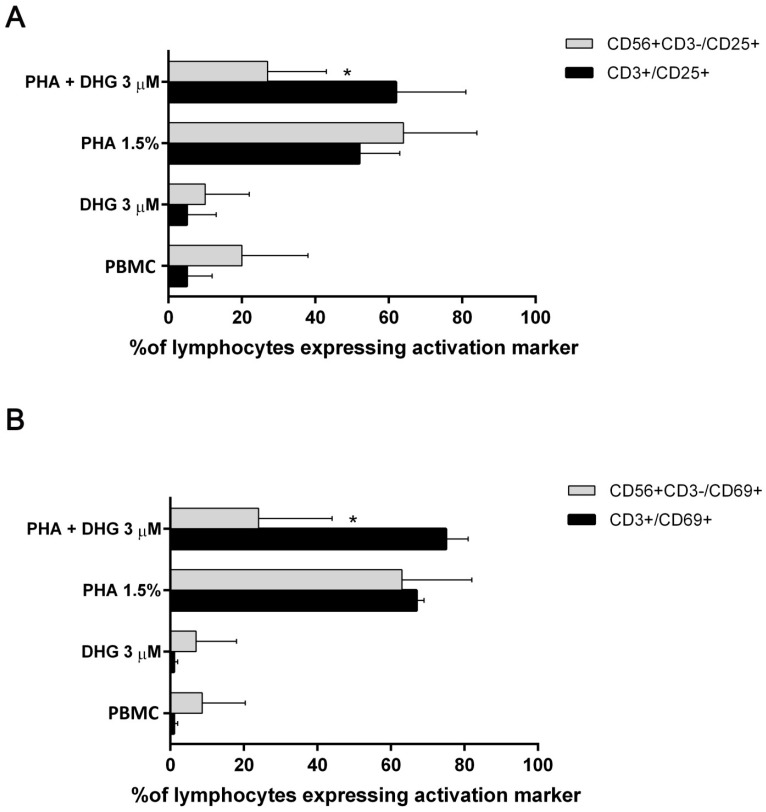
Flow cytometric analysis of CD25 and CD69 surface expression on DHG-treated PBMC. PBMC from healthy donors (*n* = 7) were stimulated with PHA (1.5%) in the presence and in the absence of DHG. Unstimulated cells are included as control (PBMC) in the figure. Following 24 h of activation, CD3+/CD56− (**black bars**) and CD3−/CD56+ (**gray bars**) populations were analyzed for CD25+ expression (**A**) and CD69+ expression (**B**) and compared by ANOVA (* *p* < 0.05, compared with untreated PHA-activated cells). Bar graphs report mean values ± SD.

All these results suggest an anti-inflammatory action of DHG. So, we asked which particular lymphocyte cell subset could be affected by DHG. In particular, we focused both on T cells (CD3+/CD56−) and natural killer (NK) cell (CD3−/CD56+) compartments. It is well known that in activation state, CD25 and CD69 are induced in lymphocytes as classical markers to monitor T and NK cell reactivity. Therefore, by fluorescence-activated cell sorting (FACS) analysis we determined the level of PBMC activation from healthy donors treated with DHG at a selected concentration (3 µM) following PHA stimulation. In samples treated with DHG compared to the control, we documented a significant decrease of the percent of CD25+ NK cells (Figure 4A) but not in T cell population. At the same way, we reported no significant difference in the surface level of CD69 in regard to the percentage of CD3+ T cells expressing the activation marker following DHG treatment, while the same compound significantly reduces the number of CD69+ NK cells (Figure 4B), suggesting a possible specific inhibition of NK cell activation that needs to be underpinned in the near future.

### 2.5. Mitogenic and Migratory Capacity of Human Keratinocytes Cell Line (HaCaT) Exposed to DHG

Then we asked if the DHG action was confined to immune compartment or if it might interfere also with proliferation and function of other cell lines. To this end, we moved to gain insight into DHG effects on keratinocyte activity, by testing its ability to modulate the viability and growth of the spontaneously immortalized human keratinocytes cell line (HaCaT) cell line, a well-established keratinocyte model, easy to propagate and near to a normal phenotype.

**Figure 5:**
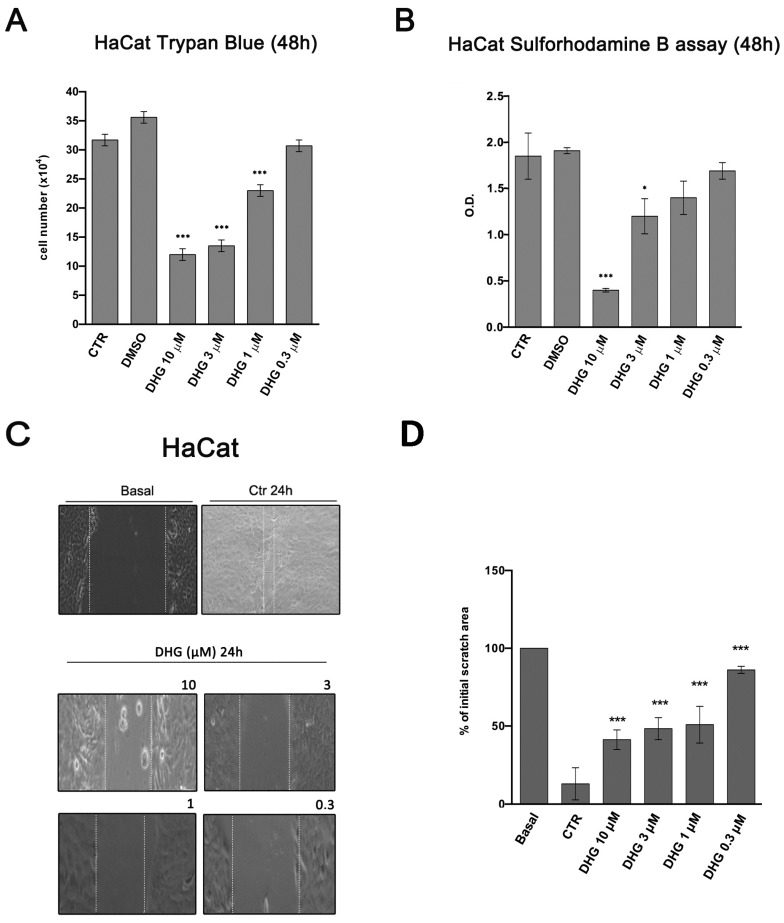
DHG effects on cell vitality and migration of HaCaT cells. (**A**) HaCaT cells were treated with vehicle alone (DMSO) or DHG at the reported concentrations, cultured for 48 h and stained with trypan blue as described in material and methods. Control cells without vehicle are also included in the figure (CTR). Cells were counted and the percent of cell viability was calculated compared to untreated cells (ANOVA * *p* < 0.05; *** *p* < 0.001); (**B**) HaCaT cells were treated with DMSO or DHG at the indicated concentrations, cultured for 48 h and next the sulforhodamine B assay was performed as described in material and methods (ANOVA * *p* < 0.05 and *** *p* < 0.001 versus DMSO-treated cells); (**C**) Wound healing assay performed in HaCaT cells treated for 24 h with vehicle (CTR) or DHG (0.3–10 µM) in complete medium. Representative light microscope images from three independent experiments are shown. Dotted white lines indicate the wounded area from the initial scratch. Magnification, × 20. Basal bar = 348.5 µm; (**D**) Histograms represent the mean scratch area observed in HaCaT cells expressed as percent of initial area. The measurement was made in three different experiments. Results are presented as mean ± standard error (ANOVA *** *p* < 0.001).

Cell counting using Trypan Blue viability dye and sulforhodamine B assay revealed respectively a reduced viability (Figure 5A) and cell growth rate (Figure 5B) of HaCaT cells exposed to 48 h treatment with increasing concentrations of DHG.

Then, to evaluate the potential interference of DHG with the migratory function of HaCaT cells, we assessed a scratch wound assay using a micropipette tip. After 24 h of cell culture, whereas serum 10% favored narrowing of the scratch wound, in the presence of DHG, the wounded cells resulted in a less enhancement of wound healing to all doses tested (Figure 5C,D).

### 2.6. Topical Anti-Inflammatory Action of DHG In Vivo

In view of the immunomodulatory potential of DHG along with its effect on hyper-activation of keratinocytes, we finally tested the marine compound in vivo in a murine model of inflammation, the croton oil ear test to evaluate its topical anti-inflammatory effect at the non-toxic dose of 1 µmol/cm^2^. This dose was selected on the basis of our experience on doses range of natural compounds active as anti-inflammatory agents in this in vivo model of skin inflammation. Interestingly, after 6 h, when oedema is already formed [59,60], DHG treatment was able to induce its significant reduction (Table 1). In particular, the oedema was decreased to about 58%, comparable to the activity of indomethacin, the reference non-steroidal anti-inflammatory drug (NSAID).

## 3. Discussion

Inflammation plays a crucial role in many physio/pathological states, and different cell populations are involved in all phases of inflammatory process, including neutrophils, dendritic cells, monocytes/macrophages, and lymphocytes. Previous findings suggested that marine diterpenoids elicit among numerous activities, anti-inflammatory effects on murine macrophages [30,31,32,33,34,35,36,37,47,53]. In this study, starting from a previous drug discovery study aimed to fractionate marine samples, we successfully demonstrated that the sponge metabolite DHG possesses promising anti-inflammatory properties in vitro and in vivo. Firstly, we assessed the ability of this natural product to reduce cell proliferation induced by different mitogens. We described efficacy of DHG to reduce dose dependently PBMC proliferation and viability (Figure 1A,B). To further investigate cellular effects of DHG, we evaluated if cell death might be caused by apoptosis. As reported in Figure 1D, we ascertained that DHG induced apoptosis in PBMC, and such effect was dose dependent as more apoptotic cells were detected at the highest concentrations of DHG. These effects are accompanied by DHG down-regulation of NF-κB, STAT and ERK phosphorylation at later time points. It is of note that the enhancement of ERK activation by 30 min treatment of DHG confirms the early apoptotic events observed (Figure 1D) and may reflect the contro-regulatory actions between these signaling events in lymphocytes biology [59]. Indeed, a prolonged and intense activation of the ERK pathway, as that in response to strong T-cell receptor (TCR) signals results in transient inhibition of IL-2-mediated activation of STAT5. In contrast, when cells receive weak signals, as that achieved by DHG low doses, the degree of activation of the ERK pathway is not strong enough to block STAT5 activation in response to small amounts of IL-2 secreted by T cells in an autocrine fashion. Our data are also in agreement with previous studies showing modulation of NF-κB, a key regulator in inflammatory processes, by several classes of marine diterpenoids in murine macrophages [35,36,37,53]. Our assays provided an initial evidence of the anti-inflammatory properties of this compound. It is well established that activated PBMC produce soluble factors that play relevant role in inflammation. Thus, cytokine secretion was assessed and the results obtained corroborate our findings. We selected three cytokines IL-6, TNF-α and IL-10. In particular, IL-6 and TNF-α are inflammatory cytokines, while IL-10 is an anti-inflammatory cytokine. The results obtained show that DHG down-regulates the expression of IL-6 that is a cytokine known to be active during inflammation, it does not affect TNF-α, thus suggesting a specific effect on particular cytokines like IL-6 and it slightly affected IL-10 secretion. The reduction of IL-10 is unexpected since it is an anti-inflammatory cytokine, however, the inhibition is very weak compared to the strong effect observed on IL-6. To establish if the effects observed on cell proliferation might be due to a reduced activation state of PBMC induced by DHG, we examined the expression of CD69, a well-known T and NKT cell early activation marker. We found that NK cell (CD3−/CD56+) compartment was affected by DHG since a reduced expression of this marker was observed. This finding is of particular interest because in agreement with previous reports describing that NK cells are protagonists of inflammatory skin diseases like psoriasis [60,61], a chronic relapsing-remitting inflammatory skin pathology characterized by thickened epidermis, as the result of keratinocyte hyper-proliferation and abnormal differentiation, increased vascularity and accumulation of inflammatory infiltrates.

Our findings are in agreement with previous studies that, in the past years, have offered suggestions that compounds from marine organisms, in particular diterpenoids, might found application in inflammatory disorders. In marine species, the ability to produce some compounds is evolutionarily selected as a substantial characteristic of defense from natural competitors. In recent years, the various properties of some natural substances, mainly of terpenoids, have made these compounds a good source of products potentially exploitable in several pharmacological applications. Published studies mainly described the effects of these molecules in murine macrophages. Here, we provided the first evidence that a diterpenoid affects also human lymphocytes. In order to investigate potential application of DHG in dermatological inflammation we analyzed its effect on immortalized keratinocytes. In these cells, we confirmed that DHG inhibits cell viability and it is also able to precociously decrease cell migration without affecting cell survival. Finally, we used an in vivo model of skin inflammation to establish a potential anti-inflammatory activity of DHG related to epidermal dysfunction. As expected, in the murine model of acute inflammation used [60,62], we observed that DHG elicited a significant anti-edema effect comparable to that of indomethacin after the induction of dermatitis.

In conclusion, our findings show that DHG reduces lymphocyte and keratinocyte proliferation and viability. In PBMC, DHG induces apoptosis reducing cell activation in a specific cell population, interferes with cytokine secretion and inhibits inflammatory pathways. In keratinocytes DHG reduces cell migration and croton oil-induced ear dermatitis in mice. Overall our results suggest a potential therapeutic use of DHG as a topical anti-inflammatory agent.

## 4. Materials and Methods

### 4.1. Reagents and Antibodies (Abs)

DHG was isolated from *D. membranosa* according to the recently described solid phase extraction (HRX-SPE) method [54]. The product (2.2 mg) was solubilized in dimethyl sulfoxide (DMSO) (0.01% in our assays) and added to cell cultures at the reported concentrations. Phytohemagglutinin (PHA) and OKT3 monoclonal antibody were from Sigma-Aldrich (St. Louis, MO, USA) and used at 1.5% and 1 µg/mL respectively.

For western blot analysis, rabbit polyclonal anti-human β-actin was purchased from Abcam (Cambridge, UK); rabbit monoclonal anti-human p-NF-κB, rabbit monoclonal anti-human NF-κB and the secondary HRP-linked antibodies were purchased from Cell Signaling Technology (Danvers, MA, USA), rabbit monoclonal anti-human p-STAT5 (Tyr 694), rabbit polyclonal anti-human STAT5, rabbit monoclonal anti-human p-p44/42 MAPK (p-ERK, Thr202/Tyr 204), rabbit monoclonal anti-human p44/42 MAPK were purchased from Cell Signaling Technology (Danvers, MA, USA); mouse monoclonal anti-human α-tubulin from Sigma-Aldrich Inc. (St. Louis, MO, USA).

The following mAbs were used for immunostaining or as blocking Abs: anti-CD56/PerCP/Cy5.5, anti-CD69/PE, anti-AnnexinV/FITC, anti-CD25 from BioLegend (San Diego, CA, USA); anti-CD3/FITC from BD Pharmingen (San Jose, CA, USA). FACSCalibur flow cytometer (BD Biosciences, San Jose, CA, USA) was used for data collection. For data analysis Cell Quest Pro program (BD Biosciences, San Jose, CA, USA) was used. Data are reported as logarithmic values of fluorescence intensity.

### 4.2. Cells

Healthy peripheral blood mononuclear cells were separated over Ficoll-Hypaque gradients (MP Biomedicals, Aurora, OH, USA). PBMC were grown in RPMI 1640 (Invitrogen, San Diego, CA, USA), supplemented with 2 mM l-glutamine, 50 ng/mL, streptomycin, 50 units/mL penicillin, and 10% heat-inactivated fetal bovine serum (Hyclone Laboratories, Logan, UT, USA). All volunteers provided written informed consent in agreement with the Declaration of Helsinki to the use of their residual buffy coats for research aims with approval from the University Hospital of Salerno Review Board.

Human immortalized keratinocytes (HaCaT) were grown in Dulbecco’s modified Eagle’s medium (DMEM, GIBCO, Grand Island, NY, USA) supplemented with 2 mM l-glutamine, 50 ng/mL, streptomycin, 50 units/mL penicillin, and 10% heat-inactivated fetal bovine serum (Hyclone Laboratories, Logan, UT, USA). HaCaT cells were kindly provided by Giuseppe Monfrecola (Department of Experimental Dermatology, University of Naples, Naples, Italy).

### 4.3. Proliferation, Cell Viability and Sulforhodamine B Assays

PBMC isolated from ten healthy donors (2 × 10^5^ cells per well) were cultured in triplicate in round bottom 96-well plates in a final volume of 200 µL of RPMI 10% FBS. Cells were activated with OKT3 or PHA. DHG was then added to the cells at the indicated concentration and its effects on proliferation were measured by the procedure described in detail elsewhere [63]. Viability of healthy PBMC (2 × 10^5^ cells per well) activated with PHA and cultured in 96-well plates in the presence and in the absence of DHG was determined by trypan blue staining and haemocytometer counting after 6 days of incubation. Unstimulated PBMC were used as control, a further control was the solvent DMSO in the presence of our stimuli (OKT3 and/or PHA). Cell viability of HaCaT cells (4 × 10^3^/well) incubated with increasing doses of DHG for 48 h was also determined by trypan blue staining and haemocytometer counting. In particular, cells were plated in 48-well plates after 24h to let them to adhere to the plastic, we added DHG at the doses indicated and after further 48h cells were detached with trypsin, stained with trypan blue and counted.

For the Sulforhodamine B assays HaCaT cells were plated in 96-well plates at a density of 2000 cells per well and after 24 h of incubation, to allow cells to adhere to the plate, DHG was added to the cells at shown concentrations. After 48 h, the supernatant was eliminated, cells were washed with PBS and finally, trichloroacetic acid (TCA) at 10% was added for one hour under stirring at 4 °C. After incubation, TCA was deleted and several washes with water were made. After drying the plates, the sulforhodamine B was added and the cells were incubated at RT for 30 min. After having eliminated the dye, washes were made with 1% acetic acid, until the removal of the unbound dye and the plates were left to dry. In the next step, the dye was solubilized with a solution of TRIS HCL 10 mM. The reading was performed at 570 nm using the spectrophotometer.

### 4.4. Scratch Wound Healing Assay

To evaluate the effect of DHG on HaCaT cell migration, the cells were plated in 6-well plates at a density of 5 × 10^3^ cells/well. After 6 days, the confluent cells formed a homogeneous carpet and a vertical wound in the wells was performed using a 200 μL tip. After a wash to remove the cells detached from the plate, culture medium containing DHG (10–0.3 µM) or the vehicle alone was added to the wells. The wound area was recorded immediately and after 24 h through microscope analysis.

### 4.5. Apoptosis Analysis

The determination of apoptosis of PBMC was conducted by Annexin V (BioLegend, San Diego, CA, USA) and PI staining. PBMC isolated from ten healthy donors (2 × 10^5^ cells per well) were cultured in a final volume of 200 µL of RPMI 10% FBS in triplicate in round bottom 96-well plates. Cells were activated with PHA (1.5%) in the presence or in the absence of increasing concentrations of DHG. After 48 h of incubation, PBMC were washed in PBS and subjected to apoptosis determination by the procedure described in detail elsewhere [64].

### 4.6. Flow Cytometric Assay

PBMC were cultured in medium, activated with PHA in the presence and in the absence of DHG 3 µM, in U-bottom 96-well plates. After 24 h, cells were washed with PBS 2% FBS, stained with anti-CD3 FITC (BD Biosciences) and anti-CD56 PE-Cy5 (BD Biosciences). The cells were acquired by flow cytometer and analyzed by Cell-Quest Pro software (BD Biosciences, San Jose, CA, USA). Results are reported as logarithmic values of fluorescence intensity.

### 4.7. Cytokine Secretion Measurement

PBMC isolated from ten healthy donors (2 × 10^5^ cells per well) were cultured in a final volume of 200 µL of complete medium in triplicate in round bottomed 96-well plates. PBMC were incubated with DHG for 24 h. Thereafter, supernatant was collected and the concentrations of TNF-α, IL-6 and IL-10 evaluated by enzyme-linked immunosorbent assay (ELISA), according to the manufacturers’ instruction (R&D Systems, Minneapolis, MN, USA; and BioSource International, Camarillo, CA, USA). Of note, the remaining cell pellets were used to analyze the surface expression of the CD3, CD69 and CD56.

### 4.8. Western Blot (WB) Analysis

PBMC (1 × 10^6^) were serum starved for 4h in T25 flasks and pre-treated with DHG at different concentrations and then activated with 1.5% PHA in sterile eppendorf for 30 min and 120 min in RPMI free medium. Then cells were centrifuged, cell pellets were lysed in ice-cold lysis buffer (20% SDS, 50% Tris, HCl 1 M, pH 6.8, 5%-ME, 25% glycerol, bromophenol blue) and then assayed for WB by the procedure described in detail elsewhere [60].

### 4.9. Topical Anti-Inflammatory Activity

DHG topical anti-inflammatory effect was reported as block of the croton oil-induced ear dermatitis in mice by the procedure described in detail elsewhere [59,60]. All the in vivo assays complied with the Italian Decree n. 116/1992 (as well as the EU Directive 2010/63/EU) and the European Convention ETS 123.

### 4.10. Statistical Analysis

In all experiments statistical analysis was performed by GraphPad prism 6.0 software for Windows (GraphPad Software Inc., La Jolla, CA, USA). Results from multiple experiments are calculated as mean ± SD and analyzed for statistical significance using the 2-tailed Student *t*-test, for independent groups, or ANOVA followed by Bonferroni correction for multiple comparisons. *p* values less than 0.05 were considered significant.

## Figures and Tables

**Table 1:** Anti-inflammatory activity of DHG in a murine model of dermatitis. Dose-dependent anti-oedema activity of topically administered DHG (1 µmol/cm^2^) and indomethacin (0.3 µmol/cm^2^) in croton oil-induced ear dermatitis after 6 h. * *p* < 0.001 at the analysis of variance, as compared to controls.

Substance	Dose (µmol/cm^2^)	Number of Animals	Edema (mg)	Reduction (%)	*p*
Controls	-	10	8.5 ± 0.2	-	-
DHG	1.0	10	3.6 ± 0.2 *	58	0.001
Indomethacin	0.3	10	3.7 ± 0.3 *	56	0.001

## Supplementary Materials

Supplementary materials can be found at www.mdpi.com/1422-0067/18/8/1643/s1.
