# Supplementary material for: Immuno-Modulatory and Anti-Inflammatory Effects of Dihydrogracilin A, a Terpene Derived from the Marine Sponge Dendrilla membranosa
Source: Int J Mol Sci. 2017 Jul 28;18(8):1643. doi: 10.3390/ijms18081643 (PMC5578033; doi:10.3390/ijms18081643)
Supplement: Supplementary file 1 [file ijms-18-01643-s001.pdf]

Supplementary data

Figure legends

Figure 1. DHG induction of apoptosis.

PBMC isolated from healthy donors (2 x 10^5 cells per well) were cultured in a final volume of 200 µl of RPMI 10% FBS in triplicate in round bottom 96-well plates in a final volume of 200 µl of RPMI 10% FBS. Cells were activated with PHA (1,5%) in the presence or in the absence of increasing concentration of DHG. After 48h of incubation, PBMC were washed in PBS and subjected to apoptosis determination. Induction of apoptosis was measured by annexin V and propidium iodide (PI) double staining through FACS analysis. The panel reports representative dot plots of 4 different experiments performed with similar results.

Figure 2. DHG effect on NF-kB phosphorylation.

PBMC isolated from healthy donors (1x10^6cells) were cultured with PHA (1,5%) and DHG in RPMI 10 % FBS for 5 days in 24-well plates. After 5 days of incubation, cells were washed with PBS, harvested and lysed in ice-cold RIPA lysis buffer (50 mM Tris-HCl, 150 mM NaCl, 0.5% Triton X-100, 0.5% deoxycholic acid, 10 mg/ml leupeptin, 2 mM phenylmethylsulfonyl fluoride, and 10 mg/ml aprotinin) and then assayed for Western blot. Western blot analysis was performed on whole cell extracts from 5 days culture of unstimulated PBMC (PBMC), PHA stimulated PBMC, PHA stimulated PBMC with DMSO in the presence and in the absence of DHG. β-actin was used as control of protein loading. Panel shows a representative result from 3 different experiments performed independently. Histograms on the right represent densitometric analysis of the obtained results.

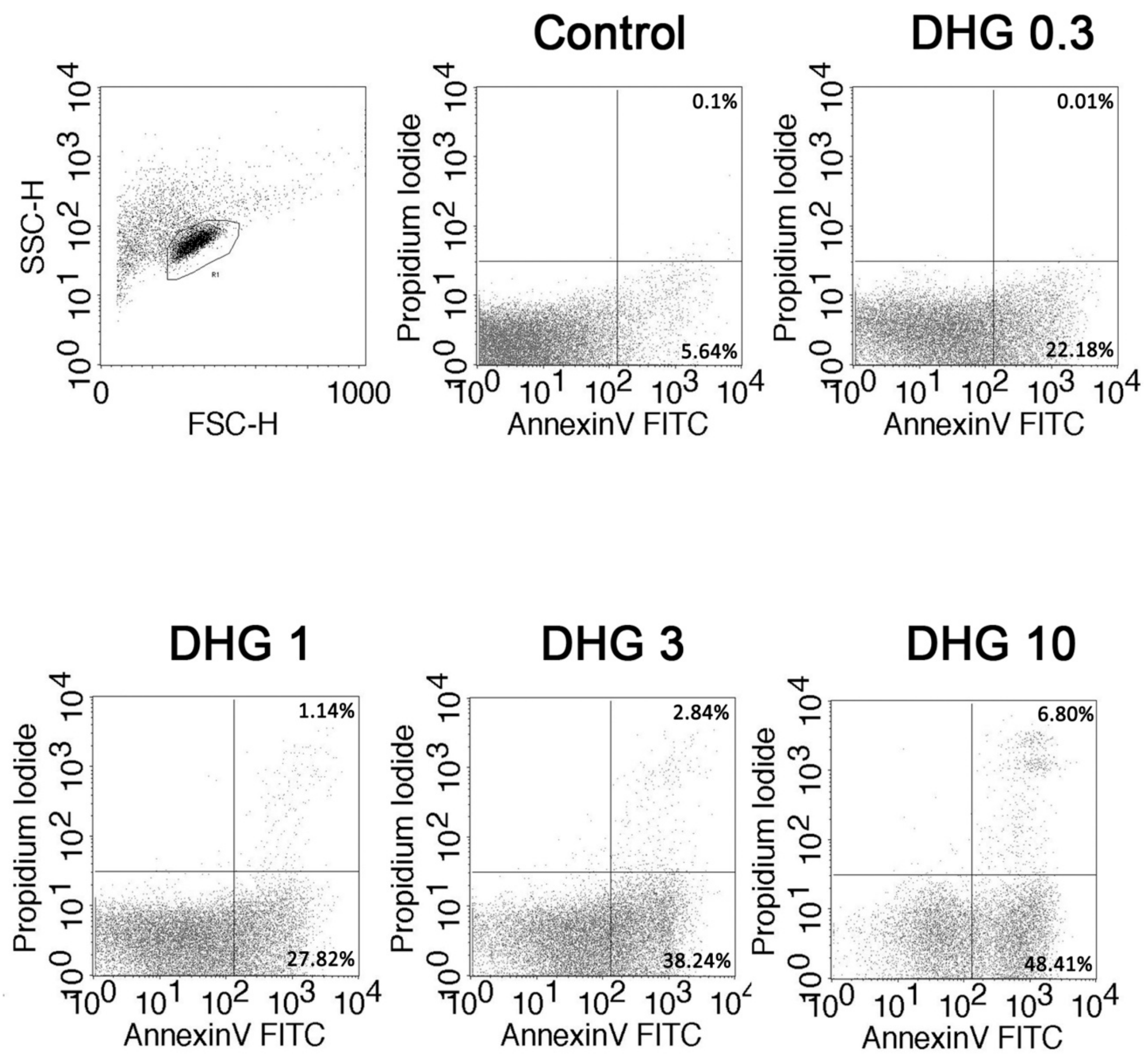

Supplementary figure 1

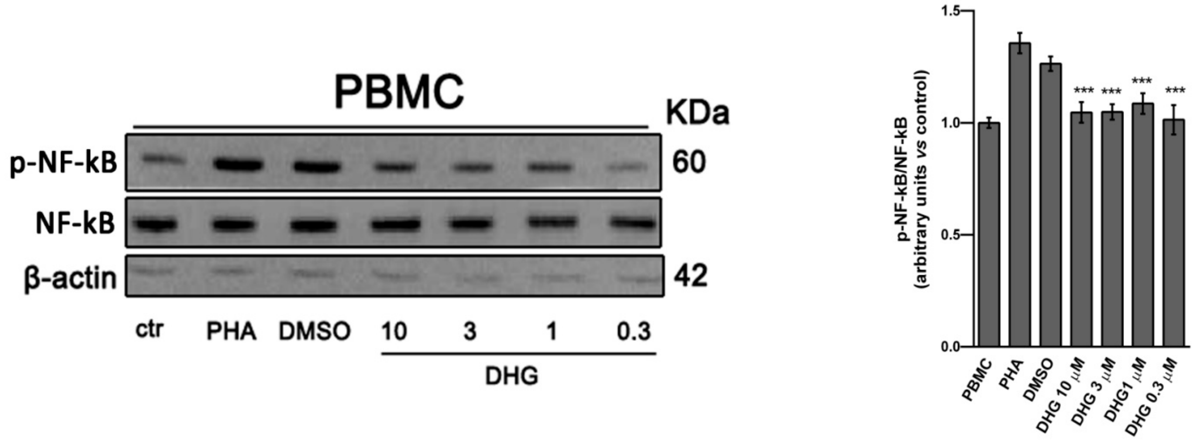

Supplementary Figure 2
